# Sparse keypoint segmentation of lung fissures: efficient geometric deep learning for abstracting volumetric images

**DOI:** 10.1007/s11548-024-03310-z

**Published:** 2025-01-07

**Authors:** Paul Kaftan, Mattias P. Heinrich, Lasse Hansen, Volker Rasche, Hans A. Kestler, Alexander Bigalke

**Affiliations:** 1 Institute of Medical Systems Biology, Ulm University, Albert-Einstein-Allee 11, 89081 Ulm, Germany; 2 Institute of Medical Informatics, University of Lübeck, Ratzeburger Allee 160, 23562 Lübeck, Germany; 3 International Graduate School in Molecular Medicine, Ulm University, Albert-Einstein-Allee 11, 89081 Ulm, Germany; 4 MoMAN Center for Translational Imaging, Ulm University, Albert-Einstein-Allee 23, 89081 Ulm, Germany; 5 EchoScout GmbH, Maria-Goeppert-Str. 3, 23562 Lübeck, Germany

**Keywords:** Pulmonary fissures, Segmentation, 3D image processing, Geometric deep learning, Point clouds, Mesh reconstruction

## Abstract

**Purpose:**

Lung fissure segmentation on CT images often relies on 3D convolutional neural networks (CNNs). However, 3D-CNNs are inefficient for detecting thin structures like the fissures, which make up a tiny fraction of the entire image volume. We propose to make lung fissure segmentation more efficient by using geometric deep learning (GDL) on sparse point clouds.

**Methods:**

We abstract image data with sparse keypoint (KP) clouds. We train GDL models to segment the point cloud, comparing three major paradigms of models (PointNets, graph convolutional networks (GCNs), and PointTransformers). From the sparse point segmentations, 3D meshes of the objects are reconstructed to obtain a dense surface. The state-of-the-art Poisson surface reconstruction (PSR) makes up most of the time in our pipeline. Therefore, we propose an efficient point cloud to mesh autoencoder (PC-AE) that deforms a template mesh to fit a point cloud in a single forward pass. Our pipeline is evaluated extensively and compared to the 3D-CNN gold standard nnU-Net on diverse clinical and pathological data.

**Results:**

GCNs yield the best trade-off between inference time and accuracy, being $21\times$ faster with only $1.4\times$ increased error over the nnU-Net. Our PC-AE also achieves a favorable trade-off, being $3\times$ faster at $1.5\times$ the error compared to the PSR.

**Conclusion:**

We present a KP-based fissure segmentation pipeline that is more efficient than 3D-CNNs and can greatly speed up large-scale analyses. A novel PC-AE for efficient mesh reconstruction from sparse point clouds is introduced, showing promise not only for fissure segmentation. Source code is available on https://github.com/kaftanski/fissure-segmentation-IJCARS

**Supplementary Information:**

The online version contains supplementary material available at 10.1007/s11548-024-03310-z.

## Introduction

3D convolutional neural networks (CNNs) are the state of the art for volumetric medical image segmentation. However, with increasing resolution of the images, the computation and memory demand grow cubically. This can make 3D-CNNs difficult to adopt in resource-constrained environments or large-scale analyses.

The lung fissures are the thin anatomical boundaries between the pulmonary lobes. Fissures can limit the spread of inflammation or neoplasia [1], making functional and pathological analysis important. Therefore, segmenting fissures is an essential task. As a thin boundary, fissures only represent 0.2 $\%$ of the volume in a thorax CT, rendering the 3D image representation highly inefficient. This shows when using the gold standard 3D-CNN segmentation framework, nnU-Net [2], for fissure segmentation, which takes 40 s on a high-performance system. This is unacceptable in large-scale post hoc analyses or opportunistic screening, where inference time is crucial.

Instead of a dense image representation, in this work, we investigate employing a sparse representation of the data: point clouds. We extract keypoints (KPs) to abstract from the volumetric image and gain a sparse point cloud that we can segment using geometric deep learning (GDL) models. We expand upon our previous work [3], where we presented a novel framework for KP-based fissure segmentation. The framework comprises KP extraction, point cloud segmentation, and fissure mesh reconstruction. We previously investigated different KP and feature extraction methods [3]. In this work, we further study segmentation and reconstruction in our pipeline. We compare three different paradigms of GDL for point cloud segmentation. We employ a PointNet [4], a GCN [5], and a PointTransformer [6]. Mesh reconstruction makes up most of the inference time in the pipeline. Therefore, we propose a novel point cloud to mesh autoencoder (PC-AE) to replace classical mesh reconstruction algorithms like Poisson surface reconstruction (PSR) [7].

Compared to the nnU-Net, our pipeline with a GCN manages a speed-up of $35\times$ (with $1.6\times$ higher surface error) or a $21\times$ speed-up at $1.4\times$ the error. We show the importance of local information exchange between points that graph convolution and attention operations provide over PointNets, which perform up to $2\times$ better. Compared to PSR, our PC-AE is $3\times$ faster through GPU-accelerated, learned mesh deformation while yielding only $1.5\times$ more error.

### Related work

Segmentation of pulmonary fissures and lobes has been performed with specially designed enhancement filters [8], shape modeling [9], and, recently, 3D-CNNs [10, 11]. These approaches have in common that their computations are performed on dense volumetric images, leading to high computational demand. In our work, we overcome this limitation by using sparse point clouds instead.

There are end-to-end approaches that generate object meshes from 3D medical images. Voxel2Mesh [12] and MeshDeformNet [13] both use a base 3D-CNN for segmentation and deform a template mesh to fit the object of interest with graph convolutions. Both use a sphere as a template, making the approaches less applicable to fissure segmentation, as fissures are open surfaces, topologically.

In a similar pipeline to ours, Balsiger et al. [14] perform peripheral nerve segmentation with a 3D-CNN and refine the segmentation with a GCN. This approach inspired our work to use a 3D-CNN for point cloud extraction. However, we choose a much more lightweight *pre*-segmentation network and promote a high recall of fissure points.

Chen et al. [15] perform point cloud-based fissure segmentation. However, they use handcrafted features and region growing for segmentation. To the best of our knowledge, we were the first to apply GDL for point-based lung fissure segmentation in our previous work [3] and expand on it here.

**Fig. 1:**
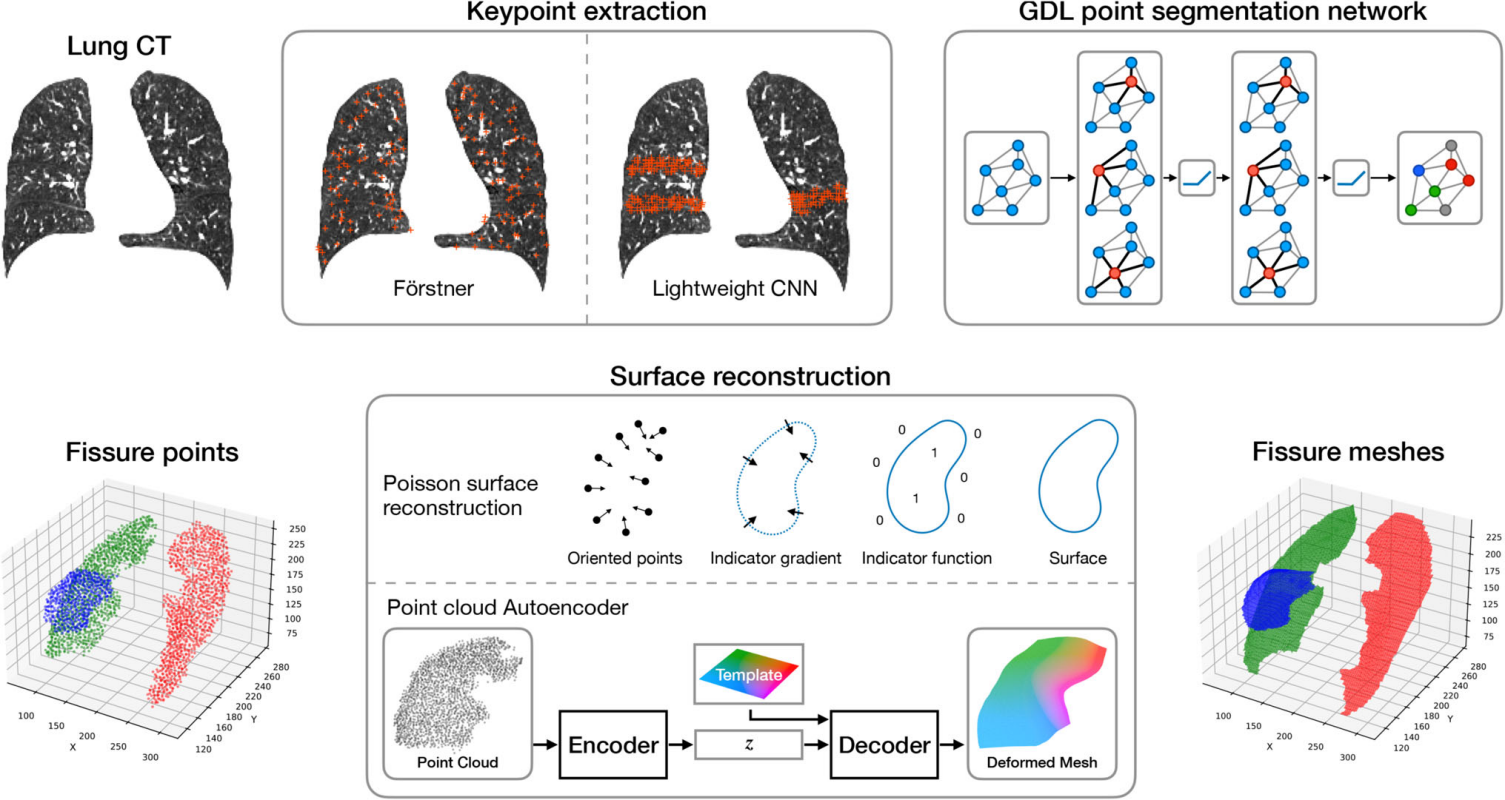
Overview of our keypoint-based fissure segmentation pipeline. We extract a sparse point cloud either in an unsupervised generic (Förstner [16]) or a supervised fissure-specific way (CNN). Then, we apply a geometric segmentation network to assign fissure labels to the points. We reconstruct a dense surface from the segmented points using Poisson surface reconstruction [7] or our point cloud autoencoder

## Materials and methods

We describe our pipeline for KP-based geometric segmentation of lung fissures from lung CT images as depicted in Fig. 1.

### Keypoint and feature extraction

First, the input CT image is abstracted into a KP cloud by selecting a tiny fraction of all voxels as fissure candidates. We employ the two best-performing methods from [3]. These are the generic Förstner KPs or the CNN-based pre-segmentation KPs. We limit all points to lie inside the lung mask and choose $K=20\,000$ points at most per image. *K* was chosen heuristically to balance point cloud resolution and segmentation efficiency. All coordinates are normalized and the resulting point cloud is $\varvec{P}\in [-1, 1]^{K\times 3}$. The point cloud $\varvec{P}$ carries shape information about the fissures. Providing image information in addition to the shape features greatly improves point segmentation [3]. Therefore, we adopt the most simple and effective method from [3], sampling $(5\times 5\times 5)$-sized patches of normalized image intensity around each point.^1^

***Förstner keypoints***

Förstner KPs [16] describe locally distinctive points in an image [16]. This operator is widely used in classical computer vision approaches. Since it is purely unsupervised and image-based, it is fissure-agnostic and does not require prior knowledge about the target structure. The KPs are detected as described in [17]. First, the distinctiveness measure is computed based on first-order gradients of the image in the structure tensor. By extracting the local maxima of distinctiveness in $(5\times 5\times 5)$ neighborhoods, this method produces a rather uniformly distributed point cloud (cf. Fig. 1). The points tend to be corners or blobs.

***CNN keypoints***

We also perform fissure-specific KP extraction using a lightweight 3D-CNN trained for pre-segmentation. This helps our method to efficiently incorporate the dense image representation. We choose MobileNetV3-Large [18] as the CNN architecture. We modify it by replacing 2D convolutional layers with 3D convolutions, keeping the kernel sizes and channel dimensions the same. For pre-segmentation, we need a high recall of fissure points in the strongly imbalanced fissure segmentation. Therefore, during training we weight the cross-entropy loss with the false negative rate per class in each batch. This pushes the segmentation toward high recall while tolerating a loss in precision, effectively resulting in an over-segmentation of the fissures. We choose *K* foreground points at random out of the predicted fissure points in the segmentation map as the KP cloud. See Online Resource 1 for more details on the network architecture and training procedure. To reduce the memory needed, we apply the network to patches of size $(128\times 128\times 128)$ with at least 50 % overlap as in [2].

### Point cloud segmentation networks

The point cloud segmentation network decides the fissure or background label for each candidate point based on the shape and image information. There are different paradigms of GDL for such networks, and we compare their applicability in the medical context. PointNets [4] can be universal function approximators for point sets [19]. However, they do not take point neighborhoods into account. Graph convolutional networks (GCNs) extend PointNets with convolutions on neighborhood graphs. This facilitates local information propagation on irregular point clouds [5]. More recently, the self-attention operator from transformer networks was adopted into point cloud processing architectures [6]. This allows for even more expressive information exchange in point neighborhoods. We choose a representative from each of the three paradigms as described in the following.

***PointNet***

PointNet [4] consists of per-point feature extraction with shared multi-layer perceptrons (MLPs) followed by a global max-pooling operation. The segmentation network then concatenates the global feature with point features and uses more MLPs to produce a point segmentation. The symmetric max-pooling function makes the network permutation-equivariant [4]. To stabilize training and since the structures of interest in the image are already roughly aligned, we omit the spatial transformer (T-Net) from PointNet.

***Dynamic Graph CNN (DGCNN)***

The DGCNN [5] is a GCN that replaces PointNet’s MLPs with the EdgeConv graph convolution while keeping the architecture very similar. EdgeConv works on points in a neighborhood, extracting edge features that get combined with local features. It uses the *k*-Nearest-Neighbor (*k*NN) graph $\mathcal {G}$, which we construct once from $\varvec{P}$ with $k=40$. Note that we ignore the image features for graph construction and that we keep $\mathcal {G}$ for all EdgeConv layers. We also omit the T-Net from DGCNN.

***PointTransformer***

The PointTransformer [6] combines the self-attention operation from Transformers with graph-based local processing. PointTransformer applies self-attention in *k*NN neighborhoods with $k=16$. For more expressive local feature extraction, vector attention instead of scalar dot-product attention is chosen [6]. A parametrized position encoding is added to the attention vector as well as the local point features. This allows the shape information to inform the attention weights and the resulting representation. The segmentation model of PointTransformer follows a U-Net structure with skip connections between a contracting and a mirrored expanding path.

***Implementation details***

Independent of the model architecture, we randomly sample $N=2048$ from the *K* points for each forward pass during training. Then, the network outputs a point segmentation from the point coordinates $\varvec{P}\in {{\,\mathrm{\mathbb {R}}\,}}^{N\times 3}$ concatenated with the features $\varvec{F}\in {{\,\mathrm{\mathbb {R}}\,}}^{N\times C}$. The loss function is the combined cross-entropy and Dice loss from [2]. Models are trained for 1000 epochs with the Adam optimizer [20], learning rate $\eta =0.001$, and weight decay of $10^{-5}$. The learning rate is successively lowered to $\eta \cdot 0.05$ in the last epoch using a cosine annealing schedule [21]. Since the networks are not translation-, rotation-, or scale-equivariant, we apply random rigid data augmentation to the coordinates in $\varvec{P}$. For inference, we run the forward pass 50 times with different random selections of *N* points. We accumulate the segmentation scores, ensuring all *K* points are segmented.

### Mesh reconstruction

The point segmentation networks output sparse point clouds representing the target objects. We need to reconstruct dense fissure surfaces to use them as lobar boundaries in downstream image analysis. The boundaries do not contain any relevant volume [1]. Therefore, topologically, we model the fissures as open surfaces with a single boundary component and construct our meshes accordingly.

#### Poisson surface reconstruction (PSR)

A state-of-the-art technique for mesh reconstruction is the PSR [7]. It solves the Poisson equation for the indicator function of the object implicitly described by a point cloud. The equation is based on point normals interpreted as samples of the indicator function gradient. See a schematic overview of PSR in Fig. 1. Point normals are estimated with principal component analysis and consistently oriented using the Open3D library^2^. PSR solves the Poisson equation on an underlying octree structure [7]. We set the octree depth hyperparameter to 6, striking a balance between the smoothness and resolution of the resulting triangle mesh. Finally, we remove triangles with vertices outside the lung mask and keep only the largest connected component of the mesh. The last step produces an open surface according to the fissure topology. Without the post-processing, PSR reconstructs a closed, watertight surface.

#### Point cloud to mesh autoencoder (PC-AE)

**Fig. 2:**
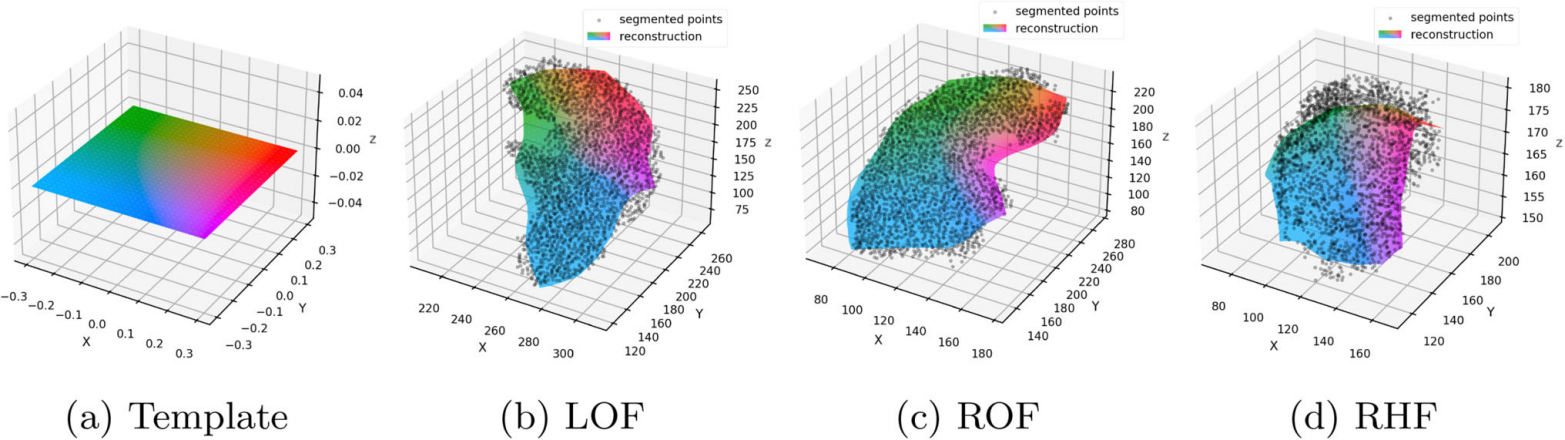
A template mesh **a** is being deformed by our point cloud autoencoder to fit an input point cloud **b**–**d**. The color coding illustrates correspondences between reconstructed meshes

Previously, PSR mesh reconstruction made up most of the inference time in our pipeline [3]. To speed this up, we propose a PC-AE for learned mesh reconstruction as shown in Fig. 1. Apart from a shorter runtime, a shape model can be an effective anatomical prior for medical deep learning [22]. The architecture is inspired by FoldingNet [23], which uses a PointNet encoder and two folding operations for decoding coordinates. We follow [24] in using a DGCNN [5] as its encoder. The global feature vector is interpreted as the latent representation. For the decoder, we found that predicting 3D deformations of a template mesh as in [12, 13] yielded much better results than the folding operation. A benefit of our approach is that we can define an initial mesh homeomorphic to our target structure. In this case, we choose the plane mesh shown in Fig. 2a. Incidentally, multiple deformed meshes contain corresponding vertices as illustrated in Fig. 2.

We adopt $N=2048$ input points and the latent vector $\varvec{z}\in {{\,\mathrm{\mathbb {R}}\,}}^h$ with $h=512$ from [23]. The template mesh to deform by the decoder is a triangle mesh with *M* vertices, *M* being the closest square number to *N*. Its vertices $\varvec{V}^{0}=(\varvec{x},\varvec{y},\varvec{0})\in \mathbb {R}^{M\times 3}$ are bilinearly sampled with $x,y\in [-0.3,0.3]$. The decoder takes *M* copies of $\varvec{z}$ in $\varvec{Z}=(\varvec{z},..., \varvec{z})^\textrm{T}\in {{\,\mathrm{\mathbb {R}}\,}}^{M\times h}$ concatenated with $\varvec{V}^{0}$. The two deforming steps are shared MLP layers $f_1, f_2:\mathbb {R}^{h+3}\rightarrow \mathbb {R}^3$ that predict residual displacements of $\varvec{V}^{0}$

$$\begin{aligned} \varvec{V}^{1}&= \varvec{V}^{0} + f_1(\varvec{Z},\varvec{V}^{0}) \\ \varvec{V}^{2}&= \varvec{V}^{1} + f_2(\varvec{Z},\varvec{V}^{1}). \end{aligned}$$

We train the PC-AE with point clouds randomly sampled from our ground truth fissure meshes. Here, we make no distinction between the three fissures so the network models the shape of all three fissures. As the training objective, we adopt the regularized mesh loss from [12] with chamfer distance (CD) as the reconstruction loss and multiple regularization terms (normal consistency (NC), edge length (EL), and Laplacian smoothness (LS)). Weights for each term are $w_\textrm{CD}=1$, $w_\textrm{NC}=0.1$, $w_\textrm{EL}=1$, and $w_\textrm{LS}=0.1$ (cf. Online Resource 1 for details). For inference in our pipeline, *N* points are sampled from an input point cloud using farthest point sampling. To reconstruct the three fissures, we perform three separate forward passes.

### Data and experiments

We choose the TotalSegmentator data set [25] for our experiments. It comprises CT images from clinical practice with various pathologies and semi-automatic segmentations, including pulmonary lobe labels. We select the 380 images that contain the lungs in their entirety. Fissure annotations are computed by finding voxels at the interface of two neighboring lobes. Ground truth fissure meshes are computed by first performing morphological binary thinning of the label maps [26, Ch. 9.5.5] and then applying PSR to the fissure voxels viewed as a point cloud with the procedure described in the “Poisson surface reconstruction (PSR)” section.

We perform a fivefold cross-validation of our pipeline in its different configurations. The results are compared to a powerful 3D-CNN trained in the nnU-Net framework [2], which is the current medical image segmentation gold standard. We choose the 3D U-Net configuration and train it for 200 epochs. To create a common fissure representation with our pipeline, meshes are reconstructed from the predicted label maps by applying binary thinning followed by PSR. Thus, we can compute surface distances between ground truth and predicted meshes. We report the average symmetric surface distance (ASSD), the standard deviation of surface distances (SDSD), and the Hausdorff distance (HD). Definitions of the metrics are given in Online Resource 1. We further validate the generalization ability of our models on a data set of COPD patients.

**Table 1:** Cross-validation of point segmentation networks compared to nnU-Net

Model	KPs	ASSD [mm]	SDSD [mm]	HD [mm]	n.a. [#]
PointNet [4]	Förstner	5.96 ± 0.65	4.80 ± 0.40	25.86 ± 1.75	**0**
	CNN	3.63 ± 0.64	3.24 ± 0.38	20.60 ± 1.80	1
DGCNN [5]	Förstner	3.54 ± 0.47	3.24 ± 0.40	20.40 ± 2.04	**0**
	CNN	3.07 ± 0.67	2.85 ± 0.38	18.37 ± 1.57	1
Point-Transformer [6]	Förstner	3.25 ± 0.45	2.95 ± 0.34	**17**.**52** ± **1**.**52**	**0**
	CNN	**3**.**01** ± **0**.**62**	**2**.**83** ± **0**.**36**	18.18 ± 1.54	2
nnU-Net [2]	–	**2**.**27** ± **0**.**85**	**2**.**50** ± **0**.**40**	**16**.**62** ± **1**.**71**	3

Bold denotes the best overall result

*ASSD* average symmetric surface distance, *SDSD* standard deviation of surface distances, *HD* Hausdorff distance.

Mesh reconstruction and surface distance computation are impossible when no keypoints are segmented for an object. We report these cases as the total number of non-assigned (n.a.) fissures

**Fig. 3:**
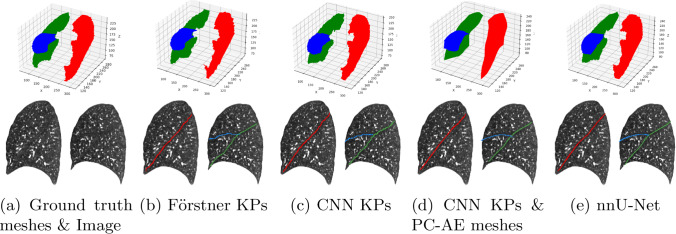
Qualitative results of our point-based pipeline with the PointTransformer **b**–**d** compared to the voxel-based nnU-Net **e**. **a** Shows ground truth meshes and the input image. Shown is case #70 from the TotalSegmentator data set. Top: reconstructed meshes with our PC-AE in **d** and PSR otherwise. Bottom left: sagittal slices of the left lung with left oblique fissure overlay (red). Bottom right: right lung with right oblique (green) and right horizontal fissure overlay (blue)

We gauge the efficiency of all parts in our pipeline by measuring the average inference time. All models, the KP extraction, and the PC-AE are implemented in PyTorch 2.2.0 and use GPU acceleration with CUDA 12.1. Our test hardware is one NVIDIA A100 80 GB GPU and an AMD EPYC 7713P CPU. Note that in our previous study [3], we measured inference times on an NVIDIA RTX 2080Ti GPU with 11 GB, which is sufficient memory to run all experiments presented here. For hardware-agnostic comparisons of the models, we also provide the number of multiply accumulate (MAC) operations per forward pass.

## Results and discussion

### Cross-validation results

Table 1 shows that PointTransformer is the strongest point segmentation network tested, followed closely by the DGCNN. The best configuration for PointTransformer with CNN KPs with 3.01 mm ASSD outperforms the DGCNN with CNN KPs at 3.07 mm. It also comes close to the gold standard nnU-Net performance with 2.27 mm. In most configurations, the pre-segmentation with CNN KPs leads to the best overall surface distances. However, in the case of PointTransformer with Förstner KPs, the HD is lower than with CNN KPs (17.52 mm vs. 18.18 mm). In this configuration, PointTransformer also outperforms DGCNN with a higher margin than with CNN KPs (3.25 mm vs. 3.54 mm ASSD). Still, both the graph convolution and the local self-attention operation are effective at leveraging feature locality in the point cloud. Comparing the PointNet with Förstner KPs to DGCNN and PointTransformer, we measure almost double the ASSD with PointNet at 5.96 mm. This suggests that local information exchange, which PointNet lacks, is especially important for the generic Förstner KPs. A major upside of Förstner over CNN KPs is that by definition of non-maximum suppression, the candidate point cloud is never empty. Thus, using Förstner KPs leads to zero missing fissures in the segmentations, making them more robust than CNN KPs (1–2 fissures missing). The nnU-Net has three missing fissures in total.

Figure 3 shows the segmentation results from PointTransformer yield visually convincing results for this example with well-contrasted fissures. See Online Resource 1 or [3, Fig. 3] for qualitative results of all models tested in this work including the best, median, and worst cases in the test data. Especially in the hard cases of abnormal appearance of the fissures, our Förstner KP-based pipeline proves to be the most robust.

Supplementary results can be found in Online Resource 1. This includes validation results of the models on COPD data. These show that our method trained on the TotalSegmentator data set generalizes well to unseen pathological data.

**Table 2:** Inference times of the different keypoint extraction methods and segmentation networks on GPU

KPs & Model	KP extr. [s]	Inference [s]	Mesh rec. [s]	Total [s]	Seg. Pts
PointNet (0.48 M par., 1.00 B MACs)					
Förstner	**0**.**10** ± **0**.**03**	**0**.**06** ± **0**.**02**	$\underline{0.95 \pm 0.05}$	**1**.**10** ± **0**.**06**	442
CNN	$\underline{0.20 \pm 0.12}$	**0**.**06** ± **0**.**02**	1.62 ± 0.11	1.89 ± 0.17	3777
DGCNN (0.65 M par., 4.36 B MACs)					
Förstner	**0**.**10** ± **0**.**03**	$\underline{0.11 \pm 0.02}$	**0**.**92** ± **0**.**04**	$\underline{1.13 \pm 0.05}$	331
CNN	$\underline{0.20\pm 0.12}$	0.12 ± 0.02	1.53 ± 0.09	1.86 ± 0.16	3283
PointTransformer (7.77 M par., 0.41 B MACs)					
Förstner	**0**.**10** ± **0**.**03**	1.18 ± 0.13	**0**.**92** ± **0**.**03**	2.20 ± 0.14	331
CNN	$\underline{0.20 \pm 0.12}$	1.32 ± 0.02	1.53 ± 0.09	3.05 ± 0.15	3272
nnU-Net (31.20 M par., 534.21 B MACs)					
	–	2.77 ± 1.12^1^	37.00 ± 15.24	39.77 ± 15.28	8845

^1^nnU-Net time measured without test-time augmentation or ensembling

PointNet [4], DGCNN [5], PointTransformer [6], and nnU-Net [2] all segment a different amount of points, influencing the duration of PSR mesh reconstruction. Bold denotes the lowest, underlining the second lowest times

### Efficiency measures

Table 2 shows that Förstner KPs are twice as fast to compute as CNN KPs (0.1 s vs. 0.2 s). Comparing the point segmentation networks with Förstner KPs, PointNet is the fastest (0.06 s), followed by DGCNN (0.11 s), and PointTransformer (1.18 s). PointTransformer has a lot more trainable parameters than DGCNN (7.77 M vs. 0.65 M) but a much lower amount of MACs (0.41 B vs. 4.36 B). Still, DGCNN inference is much faster, suggesting that the EdgeConv is more optimized on our hardware than the PointTransformer’s attention operation. Recent developments of PointTransformers [27, 28] have increased efficiency and are interesting for future work. PointNet has the lowest number of parameters at 0.48 M and MACs at 1 B, and nnU-Net has the highest (31.2 M parameters and 534.21 B MACs). The MobileNetV3 for CNN KPs uses 3.6 M parameters and 2.32 B MACs.

Mesh reconstruction time with PSR is dependent on the number of segmented points. Thus, PSR is fastest with Förstner KPs at 0.92 to 0.95 s. For CNN KPs, PSR takes between 1.53 and 1.62 s. Mesh reconstruction of the nnU-Net label maps is the slowest at 37 s, as it requires binary thinning and PSR reconstructs from the highest amount of points on average.

In total, our pipeline with PointNet and Förstner KPs is the fastest to compute at 1.1 s, but it also has the highest error (5.96 mm ASSD). Our best model in terms of ASSD, PointTransformer with CNN KPs, takes 3.05 s, and is $13\times$ faster than the nnU-Net at $1.3\times$ the error. The DGCNN with CNN KPs takes 1.86 s and is $21\times$ faster than the nnU-Net at $1.4\times$ the error. DGCNN with Förstner KPs takes 1.13 s ($35\times$ faster than nnU-Net) at $1.6\times$ the error. These trade-offs highlight the efficiency gained through point cloud sparsity compared to the voxel-based nnU-Net.

### Increasing the mesh reconstruction efficiency

***Learned mesh reconstruction with PC-AE***

PSR inference time makes up between 50 and 86 % of the runtime of our pipeline, so we replace it with a PC-AE. We selected the DGCNN as the point segmentation network, which gave us the best trade-offs between inference time and accuracy. The PC-AE takes only 0.48 s for inference instead of 1.53 s for PSR with CNN KPs ($3.2\times$ faster). This comes at the cost of decreased accuracy with 4.43 mm mean ASSD compared to 3.07 mm with PSR ($1.4\times$ higher error). The PC-AE uses 1.42 M trainable parameters and 20.24 B MACs per forward pass. To close the accuracy gap to PSR, architectural changes to the encoder or decoder or an improved optimization procedure might be necessary. Also, existing shape data sets could be leveraged to pre-train the model, increasing the shape modelling capabilities without losing any speed advantage over PSR. Still, our current results provide a strong proof of principle and warrant further investigation, which is outside the scope of this work.

With Förstner KPs, only 331 points per fissure are segmented by the DGCNN on average (cf. Table 2). This leads to a lower point density compared to the PC-AE training point clouds of size $N=2048$. The reduced density constitutes a domain shift to the DGCNN encoder as each *k*NN neighborhood now spans a larger physical area. Therefore, applying the PC-AE on these data leads to very high ASSD at 8.55 mm. However, we can diminish the impact of the domain shift by padding the input points with randomly offset ones to get *N* points in total. With this method, the ASSD becomes 4.79 mm on average. This is even faster than with CNN KPs, only taking 13.35 ms on average ($69\times$ faster than PSR at 0.92 s).

Reconstructions shown in Fig. 2 suggest that the model may be overly regularized. We performed an ablation study, of the three regularization terms (NC, EL, and LS). Removing all terms might reduced the error to 4.24 mm ASSD. However, we see that NC is crucial. Without it, the reconstructed meshes exhibit anatomically implausible foldings. Results of this ablation study and further validation on unseen COPD data are detailed in Online Resource 1.

***Mesh reconstruction from label maps***

Mesh reconstruction takes the longest for nnU-Net. We perform an ablation study to decrease the reconstruction time. Instead of applying binary thinning and considering every fissure voxel for PSR, we randomly sample 10 000 fissure points from the nnU-Net’s prediction. The number is chosen to match the number of segmented points per fissure of our pipeline with CNN KPs (cf. Table 2). This procedure drastically reduces reconstruction times to 1.6 s. With this, nnU-Net takes 4.42 ± 1.13 s in total, which is still slower than our pipeline. The fissure accuracy is slightly decreased with 2.34 ± 0.99 mm ASSD, 2.45 ± 0.45 mm SDSD, and 16.56 ± 2.01 mm HD.

***Alternative Poisson solvers***

Classical PSR [7] reconstructs fissure surfaces accurately. However, it is currently not implemented with GPU acceleration and not differentiable. Differentiable PSR (dPSR) [29] was proposed as a GPU-based alternative. In theory, this could improve our pipeline’s efficiency and enable training the point segmentation networks with mesh supervision. However, we found that the dPSR solver based on spectral methods could not fit the fissure surface. Instead, it tended more toward generating a spherical structure while ignoring more data points than classical PSR (illustration in Online Resource 1). We believe the different behavior between PSR and dPSR stems from the different underlying discretization schemes. In [7], the Poisson equation is solved on an octree, where densely sampled areas are more highly resolved than sparse regions. The dPSR solver, on the other hand, uses a fixed resolution regular grid for computing the object indicator function [29]. Thus, we speculate that the topological prior of closed surfaces influences dPSR more than PSR. For fissure surfaces, PSR still requires post-processing, excluding parts of the mesh outside the lung mask. In future work, we aim to develop an efficient and differentiable mesh reconstruction approach applicable to the fissure topology. Our PC-AE is a first step in that direction.

### Assessment of clinical impact and applicability

We have demonstrated that our point-based method can provide very efficient fissure segmentations compared to a voxel-based gold standard model (nnU-Net). The increase in efficiency could translate into practice in many scenarios. In opportunistic screening, a procedure will only be performed if it does not disrupt the main clinical workflow. Our method can be computed in only 1–2 s and is thus much more acceptable than the nnU-Net taking 40 s (cf. Table 2). In database-scale post hoc analyses, the speed advantage per scan accumulates allowing for faster results and, in turn, freeing up computational resources. Furthermore, our method natively provides a 3D mesh representation of the fissures, allowing for fast 3D visualization of the data to help treatment planning. With an error around 3 mm ASSD (cf. Table 1), our method is not as accurate as recent specialized 3D-CNNs for fissure segmentation [10, 11] at around 1 mm ASSD. However, we believe that our method provides sufficiently accurate results for a screening or visualization scenario. If more precision is required, a more resource-intensive system can still be used in a second step.

## Conclusion

We presented and extended our method for sparse keypoint (KP)-based segmentation of pulmonary fissures. Three different paradigms of geometric deep learning for keypoint segmentation were compared. Furthermore, we proposed a novel method for mesh reconstruction.

We showed that local feature extraction via graph convolution or attention is key, especially for Förstner KPs. PointNet had the fastest inference times but did not achieve satisfactory results. While the PointTransformer achieved the lowest error overall, it was $11\times$ slower to apply than DGCNN. The DGCNN has the overall best efficiency/accuracy trade-off. Compared with the 3D-CNN nnU-Net it is $21\times$ faster at $1.4\times$ the error with CNN KPs or $35\times$ faster at $1.6\times$ error with Förstner KPs. Choosing between CNN and Förstner KPs balances accuracy with robustness and efficiency.

We proposed a novel point cloud autoencoder for mesh reconstruction. It deforms a fissure-homeomorphic template mesh to fit the segmented point cloud. Reconstruction is $3\times$ faster than PSR and yields a mesh with surface correspondences over multiple objects. The error is only $1.4\times$ higher with the PC-AE.

We validated the generalization ability of our models with scans from COPD patients. In future work, we aim to test this for other pulmonary diseases such as COVID-19. Furthermore, we will work toward end-to-end differentiability of our approach.

## Supplementary Information

Below is the link to the electronic supplementary material.

Supplementary file 1 (pdf 16336 KB)

## Footnotes

1 We also investigated using patches of CNN softmax scores and Hessian fissure enhancement filter activations [8] instead. However, these features did not improve the results over patches of image intensities.

2 Documentation at https://www.open3d.org/.
