# Supplementary material for: Sparse keypoint segmentation of lung fissures: efficient geometric deep learning for abstracting volumetric images
Source: Int J Comput Assist Radiol Surg. 2025 Jan 7;20(3):465–73. doi: 10.1007/s11548-024-03310-z (PMC11929708; doi:10.1007/s11548-024-03310-z)
Supplement: Supplementary file 1 — (pdf 16336 KB) [file 11548_2024_3310_MOESM1_ESM.pdf]

Electronical Supplementary Material

Sparse Keypoint Segmentation of Lung Fissures: Efficient Geometric Deep Learning Approaches for Abstracting Volumetric Images

Paul Kaftan[1,2,3,4†], Mattias P Heinrich[2†], Lasse Hansen[5], Volker Rasche[4], Hans A Kestler[1*], Alexander Bigalke[2*]

[1]Institute of Medical Systems Biology, Ulm University, Albert-Einstein-Allee 11, Ulm, 89081, Germany.
[2]Institute of Medical Informatics, University of Lübeck, Ratzeburger Allee 160, Lübeck, 23562, Germany.
[3]International Graduate School in Molecular Medicine, Ulm University, Albert-Einstein-Allee 11, Ulm, 89081, Germany.
[4]MoMAN Center for Translational Imaging, Ulm University, Albert-Einstein-Allee 23, Ulm, 89081, Germany.
[5]EchoScout GmbH, Maria-Goeppert-Str. 3, Lübeck, 23562, Germany.

*Corresponding author(s). E-mail(s): hans.kestler@uni-ulm.de; alexander.bigalke@uni-luebeck.de;
Contributing authors: paul.kaftan@uni-ulm.de; mattias.heinrich@uni-luebeck.de; lasse@echoscout.ai; volker.rasche@uni-ulm.de;
†These authors contributed equally to this work.

Contents

1 Details on the Keypoint CNN **2**

2 Surface distance metrics **3**

3 Qualitative analysis **4**

4 Additional cross-validation results **9**

5 Validation of all models with COPD data **11**

6 Additional PC-AE experiments **16**
6.1 Hidden representation clustering 16
6.2 Ablation of regularization terms 16

7 Differentiable Poisson surface reconstruction **19**

1 Details on the Keypoint CNN

This section describes training details of the CNN model used to extract fissure-specific keypoints (KPs). We use a modified MobileNetV3 backbone with the Lite Reduced Atrous Spatial Pooling Pyramid (LR-ASPP) segmentation head [1]. The layers of the modified backbone are detailed in Table 1. The LR-ASPP head is also modified by replacing all 2D convolutional layers with their 3D counterparts of the same kernel size per dimension. In both the backbone and the head we use the leaky ReLU with negative slope 0.01 instead of the hardswish activation function. Trained models are available on GitHub.

The loss function is the cross-entropy weighted by class-specific false negative rates (FNRs). It is computed from the predicted softmax-scores per class $Y_c : \Omega \rightarrow [0, 1]$ versus the ground truth label map $L_c : \Omega \rightarrow \{0, 1\}$ for class $c \in \{0, 1, 2, 3\}$ (background and the three fissures). Both Y_c and L_c are defined over the image domain Ω. The loss function is the following:

$$\mathcal{L}_{\text{Recall}}(\boldsymbol{\theta}) = \sum_{\boldsymbol{p}\in\Omega} \sum_{c=0}^{C} -w_c \cdot L_c(\boldsymbol{p}) \cdot \ln Y_c(\boldsymbol{p}). \tag{1}$$

The predicted label map $\hat{L}_c$ is described by

$$\hat{L}_c(\boldsymbol{p}) = \begin{cases} 1, & c = \arg\max_{c' \in \{0,1,2,3\}} (Y_{c'}(\boldsymbol{p})) \\ 0, & \text{otherwise.} \end{cases} \tag{2}$$

With this, the loss weight w_c for each class can be computed as the FNR of the prediction:

$$w_c = 1 - \sum_{\boldsymbol{p}\in\Omega} \frac{\hat{L}_c(\boldsymbol{p}) \cdot L_c(\boldsymbol{p})}{L_c(\boldsymbol{p})}. \tag{3}$$

Computing the FNR on-the-fly for each minibatch describes a reliable estimate in a segmentation task, since each class is represented in many voxels.

Training of the network is performed for 50 epochs using the Adam optimizer [2] with a learning rate of 10^{-3} and weight decay of 10^{-5}. The learning rate is scheduled to decay once the validation loss does not change for a number of iterations, inspired by the nnU-Net training regime [3]. We choose a batch size of 8 and apply the network

Table 1: Modified MobileNetV3-Large [1] backbone architecture. InvRes stands for the inverted residual block. Refer to [1] for details on this operator. Conv3d are three-dimensional convolutional layers, which are followed by BatchNorm and leaky ReLU. S&E stands for Squeeze-and-Excite blocks within InvRes operators.

Input dim.	Operator	Input ch.	Expansion size	Output ch.	S&E	Stride
128^3	Conv3d, $3 \times 3 \times 3$	1	-	16	-	2
64^3	InvRes, $3 \times 3 \times 3$	16	16	16	-	1
64^3	InvRes, $3 \times 3 \times 3$	16	64	24	-	2
32^3	InvRes, $3 \times 3 \times 3$	24	72	24	-	1
32^3	InvRes, $5 \times 5 \times 5$	24	72	40	✓	2
16^3	InvRes, $5 \times 5 \times 5$	40	120	40	✓	1
16^3	InvRes, $5 \times 5 \times 5$	40	120	40	✓	1
16^3	InvRes, $3 \times 3 \times 3$	40	240	80	-	2
8^3	InvRes, $3 \times 3 \times 3$	80	200	80	-	1
8^3	InvRes, $3 \times 3 \times 3$	80	184	80	-	1
8^3	InvRes, $3 \times 3 \times 3$	80	184	80	-	1
8^3	InvRes, $3 \times 3 \times 3$	80	480	112	✓	1
8^3	InvRes, $3 \times 3 \times 3$	112	672	112	✓	1
8^3	InvRes, $5 \times 5 \times 5$	112	672	160	✓	$d = 2^*$
8^3	InvRes, $5 \times 5 \times 5$	160	960	160	✓	$d = 2^*$
8^3	InvRes, $5 \times 5 \times 5$	160	960	160	✓	$d = 2^*$
8^3	Conv3d, $1 \times 1 \times 1$	160	-	960	✓	1

*Following the Pytorch implementation of MobileNetV3, the last strided convolution with stride 2 has been replaced by subsequently dilated convolutions with $d = 2$. This achieves the same convolutional receptive field but does not reduce the spatial resolution.

on 128^3-sized image patches. During training these patches are chosen randomly. Data augmentation of each patch in a training batch comprises elastic deformations, rotation with angles sampled uniformly from $\mathcal{U}(-0.3, 0.3)$, rescaling with a factor sampled from $\mathcal{U}(0.8, 1.2)$, and a random mirroring with a chance of 35% per axis. Augmentations are implemented by the `batchgenerators` framework [4].

2 Surface distance metrics

We chose to compute three different surface distances between the ground truth and predicted meshes: average symmetric surface distance (ASSD), standard deviation of surface distances (SDSD), and Hausdorff distance (HD). All of them are based on the point-to-mesh distance d_{p2m} from point $\boldsymbol{p}$ to mesh $\mathcal{M} = (\mathcal{V}, \mathcal{F})$. $\mathcal{M}$ contains a set of vertices $\mathcal{V} \subset \mathbb{R}^3$ and a triangle list $\mathcal{F} \subset \mathcal{V}^3$. The distance equation is

$$d_{\text{p2m}}(\boldsymbol{p}, \mathcal{M}) = \min_{t \in \mathcal{F}} \{d_{\text{tri}}(\boldsymbol{p}, t)\},$$

where

$$d_{\text{tri}}(\boldsymbol{p}, t) = \min_{\boldsymbol{q} \in t} \{||\boldsymbol{p} - \boldsymbol{q}||_2\}$$

is the point-to-triangle distance, i.e. the minimum distance of $\boldsymbol{p}$ to any point $\boldsymbol{q}$ on the triangle t. From d_{p2m}, the (asymmetric) surface distances are defined as

$$\mathrm{ASSD}'(\mathcal{M}_1, \mathcal{M}_2) = \frac{1}{|\mathcal{V}_1|} \sum_{\boldsymbol{p}\in\mathcal{V}_1} d_{\mathrm{p2m}}(\boldsymbol{p}, \mathcal{M}_2)$$

$$\mathrm{SDSD}'(\mathcal{M}_1, \mathcal{M}_2) = \frac{1}{|\mathcal{V}_1| - 1} \sqrt{\sum_{\boldsymbol{p}\in\mathcal{V}_1} (d_{\mathrm{p2m}}(\boldsymbol{p}, \mathcal{M}_2) - \mathrm{ASSD}'(\mathcal{M}_1, \mathcal{M}_2))^2}$$

$$\mathrm{HD}'(\mathcal{M}_1, \mathcal{M}_2) = \max_{\boldsymbol{p}\in\mathcal{V}_1} \{d_{\mathrm{p2m}}(\boldsymbol{p}, \mathcal{M}_2)\}.$$

We make the metrics symmetric by computing the distance in both directions.

$$\mathrm{ASSD}(\mathcal{M}_1, \mathcal{M}_2) = \frac{1}{2}(\mathrm{ASSD}'(\mathcal{M}_1, \mathcal{M}_2) + \mathrm{ASSD}'(\mathcal{M}_2, \mathcal{M}_1))$$

$$\mathrm{SDSD}(\mathcal{M}_1, \mathcal{M}_2) = \frac{1}{2}(\mathrm{SDSD}'(\mathcal{M}_1, \mathcal{M}_2) + \mathrm{SDSD}'(\mathcal{M}_2, \mathcal{M}_1))$$

$$\mathrm{HD}(\mathcal{M}_1, \mathcal{M}_2) = \max\{\mathrm{HD}'(\mathcal{M}_1, \mathcal{M}_2), \mathrm{HD}'(\mathcal{M}_2, \mathcal{M}_1)\}$$

Note that for the evaluation, we only employ the symmetric distances.

3 Qualitative analysis

Figure 1 shows the test case from with the lowest ASSD measured for the DGCNN with CNN KPs. Visually, both DGCNN and PointTransformer yield convincing results. The point cloud autoencoder (PC-AE) with CNN KPs (Fig. 1k) also appears very accurate. The figure further illustrates the limitation of PointNet that lacks a local neighborhood operation, which DGCNN and PointTransformer both have. As a result, fissures segmented by PointNet in Figure 1c and 1h exhibit a non-smooth fissure surface.

Figure 2 depicts the median case of the testing set. Here, we can draw similar conclusions as from Figure 1. Notably, we see the effect of the more sparse segmented Förstner point cloud on the PC-AE reconstruction in Figure 2f. The fissure surfaces do not reach the full extent of the lung volume. In both the best and median case, the nnU-Net results are visually indistinguishable from the ground truth (Figs. 1g and Figs. 2g)

In Figure 3 we see the overall worst result of the testing set. This case has significantly shrunken parenchyma in the left lower lobe, making the LOF hard to detect. This leads to an issue in the ground truth of the LOF (Fig. 3b), which was generated from the semi-automatic TotalSegmentator annotations [5]. Only models operating on CNN KPs fail to detect LOF entirely (PointTransformer (Fig. 3j) and PC-AE (Fig. 3k)). In contrast, the non-fissure-specific Förstner KPs are more robust, providing good candidate points to segment with the GDL networks (Figs. 3c – f).

The case depicted in Figure 4 shows a case where the ROF and RHF are hard to detect. Here, the right lower lobe seems to be absent and the right middle lobe is very small in the patient. Again, the Förstner KP-based models seem to be the most

Fig. 1: Qualitative results of TotalSegmentator case s0499, which was the best result of the DGCNN with CNN KPs. Annotated is the mean ASSD result for the respective model with CNN or Förstner keypoint extraction. Top row: sagittal slices of voxelized label maps of the left oblique fissure (red). Bottom row: label maps with the right oblique (green) and right horizontal fissure (blue).

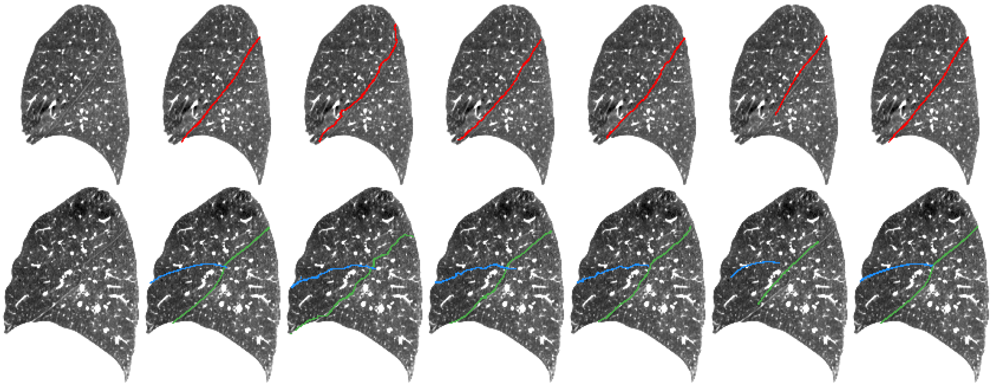

(a) Image (b) Ground truth (c) PointNet (Förstner) 4.48 mm (d) DGCNN (Förstner) 2.05 mm (e) PtTr (Förstner) 1.61 mm (f) PC-AE (Förstner) 6.45 mm (g) nnU-Net 0.76 mm

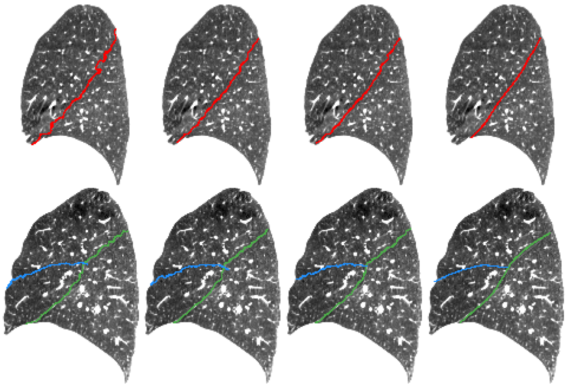

(h) PointNet (CNN) 1.83 mm (i) DGCNN (CNN) 1.15 mm (j) PtTr (CNN) 1.23 mm (k) PC-AE (CNN) 1.74 mm

capable of finding the RHF and especially the ROF (Figs. 4c – f), even compared to the nnU-Net (Fig. 4f).

We conclude from the qualitative analysis that our models with CNN KPs perform best in cases where all fissures are present and detectable (Fig. 1 and Fig. 2). Here, the pre-segmentation network can suggest proper candidate points and the resulting accuracy is better than with Förstner KPs. However, in harder cases where the fissures

Fig. 2: Qualitative results of TotalSegmentator case s1140, which was the overall median result in DGCNN with CNN KPs. Annotated is the mean ASSD result in mm for the respective model with CNN or Förstner keypoint extraction. Top row: sagittal slices of voxelized label maps of the left oblique fissure (red). Bottom row: label maps with the right oblique (green) and right horizontal fissure (blue).

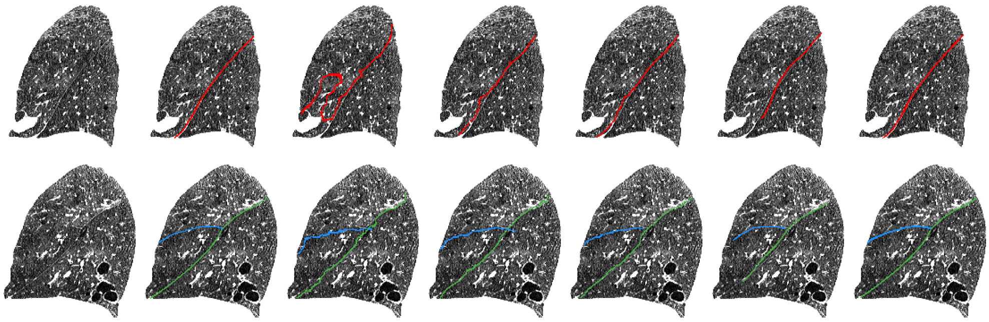

(a) Image (b) Ground truth (c) PointNet (Förstner) 4.78 mm (d) DGCNN (Förstner) 2.72 mm (e) PtTr (Förstner) 2.20 mm (f) PC-AE (Förstner) 4.13 mm (g) nnU-Net 0.77 mm

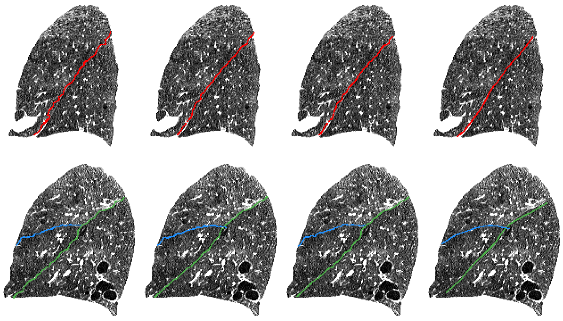

(h) PointNet (CNN) 2.37 mm (i) DGCNN (CNN) 2.00 mm (j) PtTr (CNN) 1.89 mm (k) PC-AE (CNN) 2.64 mm

do not exhibit a normal appearance, the Förstner KPs provide better results (Fig. 3 and Fig. 4). The point cloud describes an abstraction of the image appearance, such that the GDL networks can focus on segmentation by fissure shape, leading to visually more convincing results than the nnU-Net.

Fig. 3: Qualitative results of TotalSegmentator case s0650, which was the overall worst result in DGCNN with CNN keypoints. Annotated is the mean ASSD result in mm for the respective model with CNN or Förstner keypoint extraction. The LOF is not found well by all models, thus the ASSD is not assigned (n.a.). Top row: sagittal slices of voxelized label maps of the left oblique fissure (red). Bottom row: label maps with the right oblique (green) and right horizontal fissure (blue).

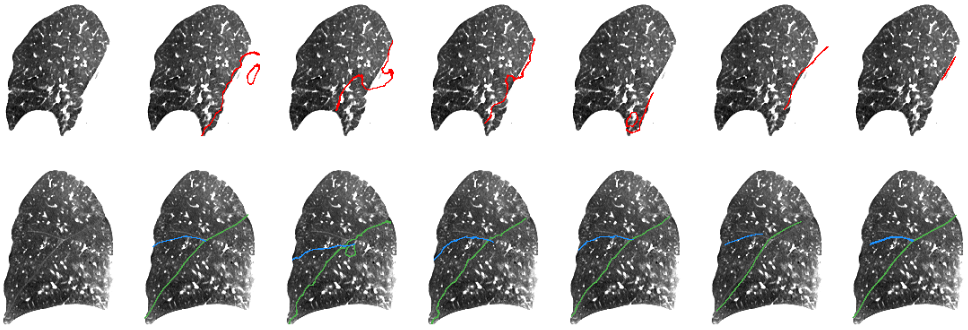

(a) Image (b) Ground truth (c) PointNet (Förstner) 8.87 mm (d) DGCNN (Förstner) 5.86 mm (e) PtTr (Förstner) 12.72 mm (f) PC-AE (Förstner) 8.12 mm (g) nnU-Net 5.89 mm

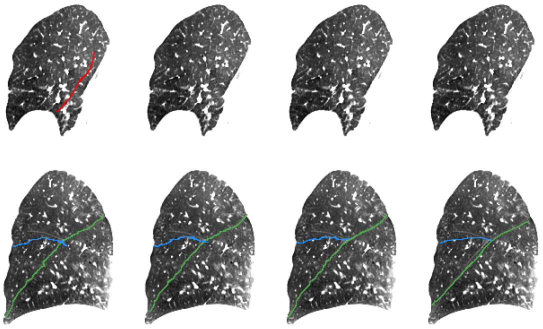

(h) PointNet (CNN) 8.79 mm (i) DGCNN (CNN) 27.92 mm (j) PtTr (CNN) n.a. (k) PC-AE (CNN) n.a.

Fig. 4: Qualitative results of TotalSegmentator case s1264, in which both right fissures were not well segmented in all models. Annotated is the mean ASSD result in mm for the respective model with CNN or Förstner keypoint extraction. Top row: sagittal slices of voxelized label maps of the left oblique fissure (red). Bottom row: label maps with the right oblique (green) and right horizontal fissure (blue).

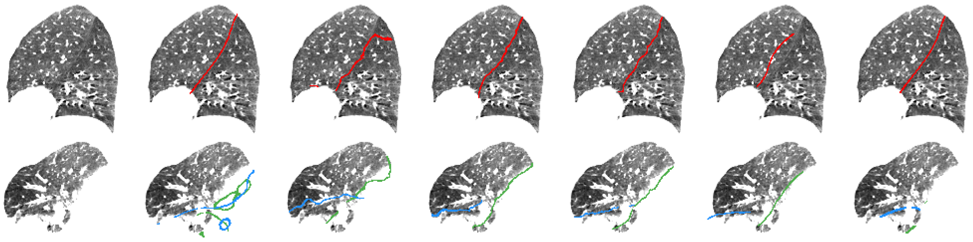

(a) Image (b) Ground truth (c) PointNet (Förstner) 17.03 mm (d) DGCNN (Förstner) 16.45 mm (e) PtTr (Förstner) 12.96 mm (f) PC-AE (Förstner) 23.82 mm (g) nnU-Net 8.50 mm

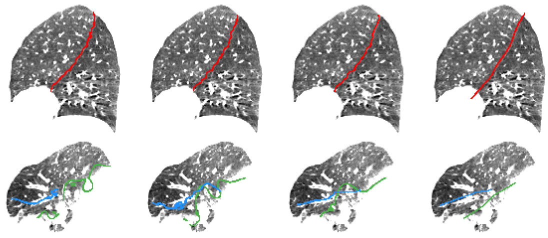

(h) PointNet (CNN) 19.98 mm (i) DGCNN (CNN) 14.85 mm (j) PtTr (CNN) 17.43 mm (k) PC-AE (CNN) 17.75 mm

4 Additional cross-validation results

Table 2 shows our cross-validation results with TotalSegmentation per class. Classes are the left oblique fissure (LOF), right oblique fissure (ROF), and right horizontal fissure (RHF). For completeness, we also report the results of the Hessian keypoint extraction method from [6].

Table 2: Cross-validation results of three point segmentation networks, PointNet [7], DGCNN [8], and PointTransformer [9] in terms of surface distances. When no keypoints are segmented for an object, mesh reconstruction is impossible and no surface distances can be computed. We report these cases as the percentage of non-assigned (n.a.) fissures.

Model	KPs	Fissure	ASSD [mm]	SDSD [mm]	HD [mm]	n.a. total
PointNet	Förstner	LOF	6.07 ± 0.46	5.43 ± 0.37	28.78 ± 1.51	0
		ROF	5.53 ± 0.75	4.85 ± 0.42	26.49 ± 1.71	0
		RHF	6.29 ± 0.74	4.13 ± 0.43	22.31 ± 2.04	0
		Mean	5.96 ± 0.65	4.80 ± 0.40	25.86 ± 1.75	**0**
	Hessian	LOF	4.42 ± 1.22	4.39 ± 0.46	27.71 ± 1.74	2
		ROF	4.14 ± 0.81	4.38 ± 0.45	27.36 ± 1.97	0
		RHF	7.36 ± 1.27	5.51 ± 0.63	28.18 ± 2.55	1
		Mean	5.31 ± 1.10	4.76 ± 0.52	27.75 ± 2.09	3
	CNN	LOF	3.42 ± 0.58	3.51 ± 0.40	22.70 ± 2.21	1
		ROF	3.33 ± 0.59	3.24 ± 0.35	21.82 ± 1.36	0
		RHF	4.15 ± 0.74	2.95 ± 0.40	17.29 ± 1.83	0
		Mean	3.63 ± 0.64	3.24 ± 0.38	20.60 ± 1.80	1
DGCNN	Förstner	LOF	3.37 ± 0.48	3.43 ± 0.42	21.01 ± 2.53	0
		ROF	3.06 ± 0.43	3.11 ± 0.43	19.68 ± 2.06	0
		RHF	4.19 ± 0.51	3.18 ± 0.35	20.51 ± 1.55	0
		Mean	3.54 ± 0.47	3.24 ± 0.40	20.40 ± 2.04	**0**
	Hessian	LOF	4.48 ± 1.46	4.31 ± 0.64	26.46 ± 2.69	2
		ROF	3.93 ± 0.81	4.19 ± 0.22	26.27 ± 1.16	1
		RHF	6.74 ± 0.61	5.52 ± 0.46	27.63 ± 1.77	7
		Mean	5.05 ± 0.96	4.67 ± 0.44	26.78 ± 1.87	10
	CNN	LOF	3.24 ± 0.91	3.19 ± 0.50	20.48 ± 2.37	1
		ROF	2.52 ± 0.61	2.69 ± 0.23	18.97 ± 0.34	0
		RHF	3.45 ± 0.50	2.68 ± 0.40	15.65 ± 1.99	0
		Mean	3.07 ± 0.67	2.85 ± 0.38	18.37 ± 1.57	1
PointTrf	Förstner	LOF	3.10 ± 0.61	3.16 ± 0.42	18.47 ± 1.73	0
		ROF	2.82 ± 0.34	2.87 ± 0.33	18.06 ± 1.42	0
		RHF	3.83 ± 0.39	2.82 ± 0.27	16.02 ± 1.41	0
		Mean	3.25 ± 0.45	2.95 ± 0.34	**17.52 ± 1.52**	**0**
	Hessian	LOF	3.93 ± 1.27	4.04 ± 0.48	25.06 ± 1.87	6
		ROF	3.62 ± 1.03	4.01 ± 0.19	25.62 ± 1.06	2
		RHF	6.77 ± 0.56	5.53 ± 0.46	27.68 ± 1.89	13
		Mean	4.77 ± 0.95	4.53 ± 0.38	26.12 ± 1.61	21
	CNN	LOF	2.98 ± 0.65	3.13 ± 0.44	20.40 ± 2.17	2
		ROF	2.58 ± 0.68	2.70 ± 0.25	18.92 ± 0.73	0
		RHF	3.46 ± 0.54	2.65 ± 0.39	15.22 ± 1.72	0
		Mean	**3.01 ± 0.62**	**2.83 ± 0.36**	18.18 ± 1.54	2
nnU-Net		LOF	2.54 ± 1.27	2.81 ± 0.43	18.18 ± 2.25	0
		ROF	1.91 ± 0.85	2.24 ± 0.36	16.22 ± 1.13	0
		RHF	2.38 ± 0.44	2.46 ± 0.41	15.47 ± 1.76	3
		Mean	**2.27 ± 0.85**	**2.50 ± 0.40**	**16.62 ± 1.71**	3

5 Validation of all models with COPD data

Since KPs are an abstraction from the image, we investigated the domain generalization ability of our pipeline compared to the dense nnU-Net prediction. We apply all trained models to the DIR-Lab COPD data set [10] and compare results to the LOF and ROF annotations from [11]. The data set comprises 20 CT scans, one inspiration, and one expiration scan each from ten patients with chronic obstructive pulmonary disease (COPD). The results for the three networks PointNet [7], DGCNN [8], and PointTransformer [9] are given in the Tables 3, 4, and 5, respectively. We report the relative surface distances from out-of-domain compared to the in-domain results to assess the generalization ability. Note that the COPD data had no annotation of the RHF, which is why we exclude the label altogether in this experiment. The tables also include results for different feature extraction methods as described previously in [6].

The ASSD measured with COPD data compared to the TotalSegmentator data set is lower with CNN KPs in all setups. For example, the DGCNN with CNN KPs and image features has 0.81× the error on the COPD data. The nnU-Net has a similar relative ASSD with a factor of 0.82×. The DGCNN with Förstner KPs and image features is slightly worse with a factor of 1.07, suggesting a higher reliance on the image information. However, we conclude from these results that our point-based segmentation method is generalizable to unseen pathological data. This robustness stems from the method being not solely reliant on image information and is helped by the fact that the TotalSegmentator training data set contains patients with diverse conditions. Still, our method does not outperform the nnU-Net in its generalization ability.

Table 6 shows the comparison between PC-AE mesh reconstruction and Poisson surface reconstruction (PSR) [12] on COPD data. Compared with PSR, the absolute surface distance metrics are worse with the PC-AE, especially for Förstner KPs (6.72 mm vs. 3.43 mm; cf. Sec. 3.3 of the main manuscript). However, the relative metrics which hint about the generalizability are better with our PC-AE. Thus, also our PC-AE model is generalizable to the unseen COPD data set.

Table 3: PointNet [7] results on COPD data. Absolute values and relative values compared to in-domain results without RHF (Tab. 2)

Keypoints	Features	Fissure	ASSD [mm]	ASSD rel.	SDSD [mm]	SDSD rel.	HD [mm]	HD rel. rel.	n.a. total
Förstner	SSC	LOF	7.28	1.04	5.08	0.95	30.09	1.06	0
		ROF	6.55	0.98	5.26	1.06	29.39	1.09	0
		Mean	6.92	1.01	5.17	1.00	29.74	1.08	0
	Image	LOF	6.02	0.99	5.39	0.99	32.24	1.12	0
		ROF	5.64	1.02	5.59	1.15	30.28	1.14	0
		Mean	5.83	1.01	5.49	1.07	31.26	1.13	0
	None	LOF	8.65	0.94	5.77	0.88	33.25	0.99	0
		ROF	7.55	0.90	5.71	0.95	31.38	1.01	0
		Mean	8.10	0.92	5.74	0.91	32.31	1.00	0
Hessian	SSC	LOF	3.26	0.80	3.96	0.91	24.89	0.92	0
		ROF	3.99	1.04	4.10	0.97	23.80	0.89	0
		Mean	3.62	0.91	4.03	0.94	24.34	0.90	0
	Image	LOF	3.36	0.76	4.08	0.93	24.49	0.88	0
		ROF	3.31	0.80	3.99	0.91	24.66	0.90	0
		Mean	3.33	0.78	4.03	0.92	24.57	0.89	0
	None	LOF	6.03	0.99	5.64	0.94	30.11	0.91	0
		ROF	5.32	1.07	5.51	1.05	30.32	1.01	0
		Mean	5.67	1.02	5.57	0.99	30.21	0.96	0
CNN	SSC	LOF	2.88	0.81	2.80	0.82	20.27	0.90	0
		ROF	3.23	0.97	3.11	1.01	20.36	0.97	0
		Mean	3.06	0.89	2.96	0.91	20.32	0.93	0
	Image	LOF	2.96	0.87	3.20	0.91	22.00	0.97	0
		ROF	2.99	0.90	3.09	0.95	20.49	0.94	0
		Mean	2.97	0.88	3.15	0.93	21.25	0.95	0
	None	LOF	3.27	0.75	2.94	0.80	20.15	0.86	1
		ROF	3.54	0.89	3.27	0.95	20.98	0.94	0
		Mean	3.40	0.82	3.11	0.87	20.56	0.90	1
nnU-Net		LOF	1.97	0.78	2.67	0.95	18.44	1.01	0
		ROF	1.68	0.88	2.09	0.93	14.05	0.87	0
		Mean	1.83	0.82	2.38	0.94	16.24	0.94	0

Table 4: DGCNN [8] results on COPD data. Absolute values and relative values compared to in-domain results without RHF (Tab. 2)

Keypoints	Features	Fissure	ASSD [mm]	ASSD rel.	SDSD [mm]	SDSD rel.	HD [mm]	HD rel. rel.	n.a. total
Förstner	SSC	LOF	4.77	1.03	4.23	1.02	27.65	1.15	0
		ROF	4.84	1.13	4.50	1.23	25.98	1.19	0
		Mean	4.80	1.08	4.36	1.12	26.82	1.17	0
	Image	LOF	3.26	0.97	3.76	1.10	26.03	1.24	0
		ROF	3.61	1.18	4.13	1.33	24.97	1.27	0
		Mean	3.43	1.07	3.94	1.20	25.50	1.25	0
	None	LOF	7.28	0.93	4.85	0.84	29.79	0.99	0
		ROF	6.57	0.91	5.12	0.99	29.19	1.06	0
		Mean	6.93	0.92	4.99	0.91	29.49	1.02	0
Hessian	SSC	LOF	2.73	0.66	3.55	0.87	22.73	0.89	0
		ROF	3.09	0.84	3.46	0.88	20.75	0.83	0
		Mean	2.91	0.75	3.51	0.88	21.74	0.86	0
	Image	LOF	2.80	0.62	3.45	0.80	20.65	0.78	0
		ROF	2.54	0.65	3.28	0.78	20.82	0.79	0
		Mean	2.67	0.64	3.36	0.79	20.73	0.79	0
	None	LOF	3.20	0.71	3.75	0.84	22.24	0.82	0
		ROF	3.24	0.92	4.00	1.00	23.51	0.93	0
		Mean	3.22	0.80	3.88	0.92	22.88	0.87	0
CNN	SSC	LOF	2.39	0.70	2.72	0.86	20.60	0.98	0
		ROF	2.49	0.95	2.65	0.96	18.08	0.94	0
		Mean	2.44	0.81	2.69	0.91	19.34	0.96	0
	Image	LOF	2.30	0.71	2.70	0.85	20.33	0.99	0
		ROF	2.36	0.94	2.63	0.98	18.19	0.96	0
		Mean	2.33	0.81	2.67	0.91	19.26	0.98	0
	None	LOF	2.87	0.75	3.02	0.90	21.34	0.97	0
		ROF	2.73	0.88	2.72	0.91	18.12	0.89	0
		Mean	2.80	0.81	2.87	0.90	19.73	0.93	0
nnU-Net		LOF	1.97	0.78	2.67	0.95	18.44	1.01	0
		ROF	1.68	0.88	2.09	0.93	14.05	0.87	0
		Mean	1.83	0.82	2.38	0.94	16.24	0.94	0

Table 5: PointTransformer [9] results on COPD data. Absolute values and relative values compared to in-domain results without RHF (Tab. 2)

Keypoints	Features	Fissure	ASSD [mm]	ASSD rel.	SDSD [mm]	SDSD rel.	HD [mm]	HD rel. rel.	n.a. total
Förstner	SSC	LOF	3.97	1.05	3.95	1.13	26.43	1.33	0
		ROF	4.09	1.19	3.77	1.20	22.95	1.19	0
		Mean	4.03	1.12	3.86	1.16	24.69	1.26	0
	Image	LOF	3.22	1.04	3.87	1.22	26.01	1.41	0
		ROF	3.42	1.21	3.94	1.37	24.52	1.36	0
		Mean	3.32	1.12	3.90	1.29	25.26	1.38	0
	None	LOF	7.47	0.99	4.80	0.87	30.39	1.06	0
		ROF	6.99	0.93	5.21	0.96	28.43	1.00	0
		Mean	7.23	0.96	5.00	0.91	29.41	1.03	0
Hessian	SSC	LOF	3.61	0.91	4.11	0.99	23.27	0.93	0
		ROF	2.68	0.76	3.34	0.85	19.89	0.80	0
		Mean	3.14	0.84	3.72	0.92	21.58	0.86	0
	Image	LOF	2.73	0.69	3.49	0.86	21.95	0.88	0
		ROF	2.50	0.69	3.20	0.80	19.32	0.75	1
		Mean	2.61	0.69	3.34	0.83	20.63	0.81	1
	None	LOF	3.31	0.85	3.91	0.95	21.93	0.88	0
		ROF	3.01	0.86	3.76	0.96	21.83	0.88	0
		Mean	3.16	0.85	3.83	0.96	21.88	0.88	0
CNN	SSC	LOF	2.42	0.81	2.75	0.88	20.51	0.98	0
		ROF	2.36	0.90	2.53	0.94	17.46	0.90	0
		Mean	2.39	0.85	2.64	0.90	18.99	0.95	0
	Image	LOF	2.34	0.79	2.79	0.89	21.00	1.03	0
		ROF	2.39	0.93	2.70	1.00	18.55	0.98	0
		Mean	2.37	0.85	2.75	0.94	19.78	1.01	0
	None	LOF	2.86	0.74	2.98	0.89	21.53	0.99	0
		ROF	2.80	0.89	2.75	0.91	18.53	0.91	0
		Mean	2.83	0.81	2.87	0.90	20.03	0.95	0
nnU-Net		LOF	1.97	0.78	2.67	0.95	18.44	1.01	0
		ROF	1.68	0.88	2.09	0.93	14.05	0.87	0
		Mean	1.83	0.82	2.38	0.94	16.24	0.94	0

Table 6: Results of PC-AE mesh reconstruction compared to PSR on COPD data. Absolute values and relative values compared to in-domain results without RHF. The point segmentation is performed using DGCNN with image features.

Keypoints	Mesh rec.	Fissure	ASSD [mm]	ASSD rel.	SDSD [mm]	SDSD rel.	HD [mm]	HD rel. rel.	n.a. total
Förstner	PSR	LOF	3.26	0.97	3.76	1.10	26.03	1.24	0
		ROF	3.61	1.18	4.13	1.33	24.97	1.27	0
		Mean	3.43	1.07	3.94	1.20	25.50	1.25	0
	PC-AE	LOF	6.34	0.72	4.81	0.71	28.74	0.76	0
		ROF	7.10	0.92	5.05	0.85	27.31	0.86	0
		Mean	6.72	0.82	4.88	0.78	28.02	0.81	0
CNN	PSR	LOF	2.30	0.71	2.70	0.85	20.33	0.99	0
		ROF	2.36	0.94	2.63	0.98	18.19	0.96	0
		Mean	2.33	0.81	2.67	0.91	19.26	0.98	0
	PC-AE	LOF	3.14	0.67	3.11	0.78	21.96	0.89	0
		ROF	3.41	0.80	2.86	0.78	18.91	0.82	0
		Mean	3.32	0.74	2.99	0.78	20.44	0.86	0

6 Additional PC-AE experiments

6.1 Hidden representation clustering

The PC-AE is trained to model all three different pulmonary fissures. We assume for the method to work that the latent space is partitioned to separate the embeddings of different fissure classes. To validate this assumption, we perform a cluster analysis of the latent space on the cross-validation test data. We take KPs segmented with a DGCNN and input them into the encoder of the PC-AE. Then, the latent embeddings are clustered by k-means clustering with $k = 3$, since we expect three fissure clusters.

In this experiment, 99.0 % of fissures were classified correctly when using CNN KPs. With Förstner KPs, 98.5 % of fissures were correctly identified. A visualization of the latent space in Figure 5 shows the separation of the three fissures. Thus, we can conclude that the latent embedding of the fissure shapes contains sensible partitioning per fissure classes.

6.2 Ablation of regularization terms

In the training of the PC-AE, we use three different regularization terms. Normal consistency (NC), edge length (EL), and Laplacian smoothness (LS) are used as implemented in [13]. NC penalizes sharp bends or foldings of the mesh that are not anatomically plausible. EL regularization encourages the length of edges in the triangle mesh to be small, resulting in a more uniform size of all triangles. LS encourages a smoother mesh matching the generally smooth shape of the fissure.

To see why the results from our PC-AE may be over-regularized, we performed an ablation study of the regularization terms. We use all possible combinations of the three terms with either their weight as defined in Section 2.3.2 of the main manuscript or 0. Results are given in Table 7. While the using no regularization has the lowest ASSD at 4.24 mm, the reconstructed mesh contains irregular foldings as illustrated in Figure 6b. These foldings are prevented in experiments where NC is used for regularization. Foregoing EL regularization leads to a non-uniform mesh resolution (Fig. 6c) and an error of 4.37 mm. Without LS some sharper edges at the border of the mesh can be seen in Figure 6d, while the overall error is 4.43 mm.

To conclude this ablation study, we do not recommend removing the regularization terms. While using no regularization may result in lower ASSD, it also leads to foldings in the meshes and a spatially non-uniform mesh resolution. This means that points on the meshes are no longer corresponding between two cases. In future work we will need to explore the training and the architecture of the PC-AE to combat lower accuracy of the meshes compared to PSR.

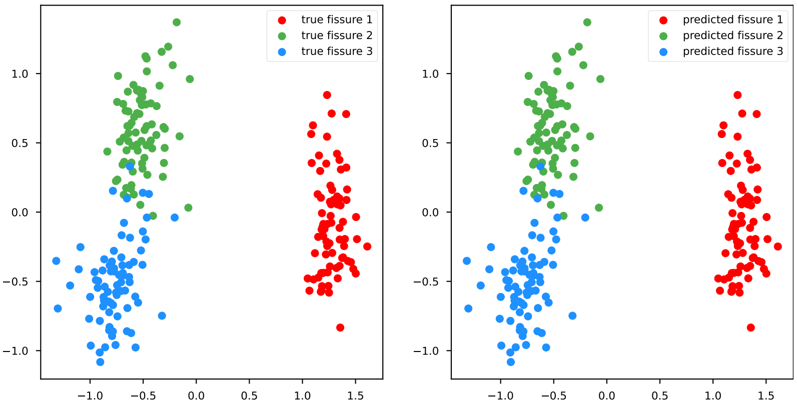

(a) Latent representations from segmented CNN KP clouds. In this testing fold, 0 misclassifications were found.

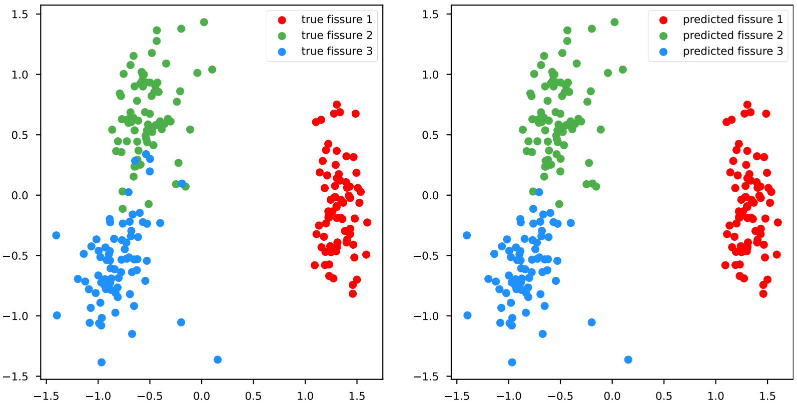

(b) Latent representations from segmented Förstner KP clouds. In this testing fold, 6 misclassifications were found.

Fig. 5: The first two principal components of the hidden representations with their actual fissure label (left) and the labels predicted by k-means clustering with $k = 3$ (right). Both CNN KPs (a) and Förstner KPs (b) yield few misclassifications.

Table 7: Ablation study of the regularization terms normal consistency (NC), edge length (EL), and Laplacian smoothness (LS) used for training the point cloud autoencoder (PC-AE). The given results are the reconstruction ASSD with the PC-AE from CNN keypoints segmented by a DGCNN. The last row corresponds to the default settings as used in the main manuscript. Weights for each term are either $w_{\mathrm{NC}} = 0.1$, $w_{\mathrm{EL}} = 1$, and $w_{\mathrm{LS}} = 0.1$ (✓) or 0 (–).

NC	EL	LS	mean ASSD [mm]
–	–	–	4.24
–	–	✓	4.25
–	✓	–	4.45
–	✓	✓	4.44
✓	–	–	4.41
✓	–	✓	4.37
✓	✓	–	4.43
✓	✓	✓	4.43

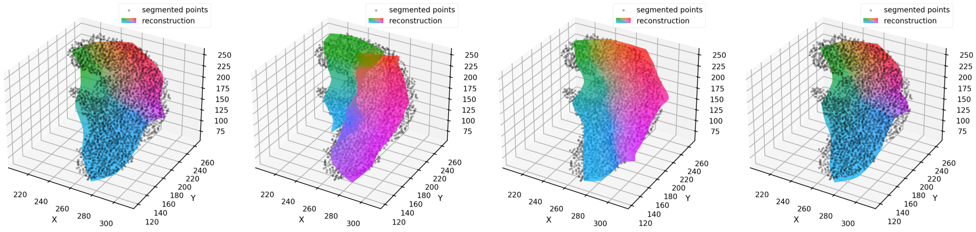

(a) NC, EL, LS (b) EL, LS (c) NC, LS (d) NC, EL

Fig. 6: Left oblique fissure reconstruction by the PC-AE with different regularization terms. (a) All three terms are used as given in the main manuscript. (b) When normal consistency (NC) is unused, the reconstructed mesh exhibits implausible folding. (c) Omitting edge length (EL) or (d) Laplacian smoothness (LS) regularization yields visually similar but less smooth results than (a).

7 Differentiable Poisson surface reconstruction

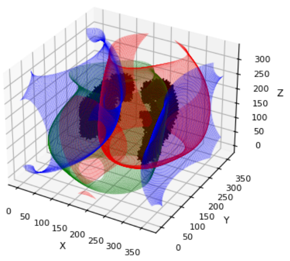

Fig. 7: Problem of the dPSR [14] solver trying to enclose the fissure point cloud.

References

[1] Howard, A., Sandler, M., Chen, B., Wang, W., Chen, L.-C., Tan, M., Chu, G., Vasudevan, V., Zhu, Y., Pang, R., Adam, H., Le, Q.: Searching for mobilenetv3. In: 2019 IEEE/CVF Int Conf Comput Vis (ICCV), pp. 1314–1324 (2019). https://doi.org/10.1109/ICCV.2019.00140

[2] Kingma, D.P., Ba, J.: Adam: A method for stochastic optimization. In: Bengio, Y., LeCun, Y. (eds.) 3rd International Conference on Learning Representations, ICLR 2015, Conference Track Proc. (2015). http://arxiv.org/abs/1412.6980

[3] Isensee, F., Jäger, P.F., Kohl, S.A.A., Petersen, J., Maier-Hein, K.H.: Automated design of deep learning methods for biomedical image segmentation. Nat Methods **18**(2), 203–211 (2021) https://doi.org/10.1038/s41592-020-01008-z

[4] Isensee, F., Jäger, P., Wasserthal, J., Zimmerer, D., Petersen, J., Kohl, S., Schock, J., Klein, A., Roß, T., Wirkert, S., Neher, P., Dinkelacker, S., Köhler, G., Maier-Hein, K.: Batchgenerators - a Python Framework for Data Augmentation. Zenodo (2020). https://doi.org/10.5281/zenodo.3632567

[5] Wasserthal, J., Meyer, M., Breit, H.-C., Cyriac, J., Yang, S., Segeroth, M.: TotalSegmentator: robust segmentation of 104 anatomical structures in CT images (arXiv:2208.05868) (2022) https://doi.org/10.48550/arXiv.2208.05868 arxiv:2208.05868 [cs, eess]

[6] Kaftan, P., Heinrich, M.P., Hansen, L., Rasche, V., Kestler, H.A., Bigalke, A.: Abstracting Volumetric Medical Images with Sparse Keypoints for Efficient Geometric Segmentation of Lung Fissures with a Graph CNN. In: Bildverarbeitung Für Die Medizin 2024. Springer, Erlangen (2024)

[7] Charles, R.Q., Su, H., Kaichun, M., Guibas, L.J.: Pointnet: Deep learning on point sets for 3d classification and segmentation. In: 2017 IEEE Conf Comput Vis Pattern Recognit (CVPR), pp. 77–85 (2017). https://doi.org/10.1109/CVPR.2017.16

[8] Wang, Y., Sun, Y., Liu, Z., Sarma, S.E., Bronstein, M.M., Solomon, J.M.: Dynamic graph CNN for learning on point clouds. ACM Trans Graph (2019)

[9] Zhao, H., Jiang, L., Jia, J., Torr, P., Koltun, V.: Point Transformer. In: 2021 IEEE/CVF Int Conf Comput Vis (ICCV), pp. 16239–16248 (2021). https://doi.org/10.1109/ICCV48922.2021.01595

[10] Castillo, R., Castillo, E., Fuentes, D., Ahmad, M., Wood, A.M., Ludwig, M.S., Guerrero, T.: A reference dataset for deformable image registration spatial accuracy evaluation using the copdgene study archive. Phys Med Biol **58**(9), 2861–2877 (2013) https://doi.org/10.1088/0031-9155/58/9/2861

[11] Rühaak, J., Polzin, T., Heldmann, S., Simpson, I.J.A., Handels, H., Modersitzki, J., Heinrich, M.P.: Estimation of large motion in lung CT by integrating regularized keypoint correspondences into dense deformable registration. IEEE Trans Med Imaging **36**(8), 1746–1757 (2017) https://doi.org/10.1109/tmi.2017.2691259

[12] Kazhdan, M., Hoppe, H.: Screened poisson surface reconstruction. ACM Trans Graph **32**(3), 1–13 (2013) https://doi.org/10.1145/2487228.2487237

[13] Ravi, N., Reizenstein, J., Novotny, D., Gordon, T., Lo, W.-Y., Johnson, J., Gkioxari, G.: Accelerating 3D deep learning with PyTorch3D. arXiv:2007.08501 (2020) arXiv:2007.08501

[14] Peng, S., Jiang, C., Liao, Y., Niemeyer, M., Pollefeys, M., Geiger, A.: Shape as points: A differentiable poisson solver. In: Ranzato, M., Beygelzimer, A., Dauphin, Y., Liang, P.S., Vaughan, J.W. (eds.) Adv Neural Inf Process Syst (NeurIPS 2021), vol. 34, pp. 13032–13044. Curran Associates, Inc., Red Hook, NY (2021)
